# Temporal trends of migraine and tension-type headache burden across the BRICS: implications from the Global Burden of Disease study 2019

**DOI:** 10.3389/fneur.2023.1307413

**Published:** 2023-12-22

**Authors:** Yuan-jie Zhang, Xin-yu Li, Zhi-lin Guo

**Affiliations:** ^1^Department of Neurosurgery, Shanghai Ninth People’s Hospital, Shanghai Jiao Tong University, Shanghai, China; ^2^Department of Plastic and Reconstructive Surgery, Shanghai Ninth People’s Hospital, Shanghai Jiao Tong University School of Medicine, Shanghai, China

**Keywords:** Global Burden of Disease, migraine, tension-type headache, TTH, GBD

## Abstract

**Background:**

Headache disorders have become a significant global public health issue, with a notably high prevalence observed in developing countries. However, few studies have assessed headache disorders trends in Brazil, Russia, India, China and South Africa (BRICS). This study aimed to assess the prevalence of headache disorders in individuals across the BRICS, spanning the years 1990 to 2019.

**Methods:**

We obtained headache disorders data from the Global Burden of Disease 2019 study (GBD2019). This evaluation examined incidence rates, prevalence, and disability-adjusted life-years (DALYs) for migraine and tension-type headache (TTH) across demographic factors like age, gender, year, and country. Migraine and TTH were diagnosed based on the International Classification of Headache Disorders (ICHD-3) criteria. We used disease codes from the International Classification of Diseases, 10th revision to identify migraine and TTH cases. Statistical analyzes included calculating age-standardized rates and estimated annual percentage changes. Future disease burden was projected using a log-linear age-period-cohort model.

**Results:**

In 2019, India had the highest prevalence of migraine (213890207.93 cases) and TTH (374,453,700 cases). Brazil had the highest migraine age-standardized prevalence rate (18,331 per 100,000) and incidence rate (1,489 per 100,000). For TTH, India had the highest prevalence (26,160 per 100,000) while Russia had the highest incidence (11,512 per 100,000). From 1990 to 2019, China showed the greatest increase in migraine and TTH prevalence. India had the highest migraine (7,687,692) and TTH (741,392) DALYs in 2019.

**Conclusion:**

Migraine and TTH remain highly prevalent in BRICS nations, inflicting considerable disability burden. While India and China face mounting disease prevalence, Brazil contends with high incidence rates. Tailored interventions based on country-specific epidemiological profiles are warranted to mitigate the public health impact.

## Introduction

Headache disorders, encompassing a spectrum of neurological ailments, are among the most prevalent and incapacitating conditions, affecting nearly half of the global adult population (1). The most common forms of primary headaches are migraines and tension-type headaches (TTH) (2). Migraines, which exhibit a global age-standardized prevalence of approximately 15% and show a pronounced predilection for women over men, are distinct from TTH, with the latter boasting a prevalence of 42% in the global population and carrying significant socio-economic repercussions (3–5). Both migraine and TTH can significantly impair quality of life and cause substantial disability and lost productivity (6). Beyond the individual pain and distress, both conditions collectively contribute to considerable socio-economic implications. Migraine ranks as the second leading cause of years lived with disability globally (7). TTH also imposes a substantial health burden, accounting for about one-third of all headache-attributed disability (8, 9).

Brazil, Russia, India, China, and South Africa, denoted collectively as the BRICS nations, encompass approximately half of the world’s populace and are distinguished by their burgeoning economies and ascendant economic stature (10). Recent analyzes highlight the significant societal implications of migraine and TTH within the BRICS countries, rooted in their surging prevalence, notable disability, and substantial economic burdens (11, 12). However, in stark contrast, headache disorders consistently remain under-identified and insufficiently addressed within these populations. Recognizing the burgeoning global prominence of these nations, it becomes imperative to meticulously characterize and address the inherent health challenges they face. Notwithstanding their rising profile in global health discourse, there is a discernible lack of detailed epidemiological insights into afflictions such as migraine and TTH within these regions. To surmount this deficiency, our study leveraged the Global Burden of Disease (GBD) database, examining trends in incidence, prevalence, and Disability Adjusted Life Years (DALYs) related to headache disorders in the BRICS nations from 1990 to 2019. Furthermore, we project the future disease burden. Our intent is to offer a comprehensive analysis of the epidemiology of migraine and TTH within the context of the BRICS nations, providing public health officials with insights to evaluate the outcomes of prior interventions and to shape subsequent policy directives.

## Methods

### Data sources

We obtained the population, prevalence, incidence, and age-standardized rates (ASR) data for all age and age-standardized groups across the BRICS from GBD 2019. This dataset is accessible at http://ghdx.healthdata.org/gbd-results-tool (accessed on 2023-06-07). The GBD 2019 amassed information relevant to 369 diseases and injuries and scrutinized 87 associated risk factors spanning 204 countries and territories (13). We conducted our study in strict adherence to the Strengthening the Reporting of Observational Studies in Epidemiology (STROBE) guidelines. Given its cross-sectional design, our study received ethical clearance from the Shanghai Ninth People’s Hospital. The board provided a waiver for informed consent, considering our exclusive focus on data analysis and the absence of personal identifying information.

The GBD aggregated data from a plethora of sources, encompassing censuses, surveys, vital statistics, and a myriad of health databases. To adeptly synthesize this extensive dataset, the GBD model utilizes three advanced methodologies: the Cause of Death Ensemble model, the spatiotemporal Gaussian process regression, and the Bayesian meta-regression tool, DisMod-MR 2.1 (14).

### Disease definition

Within the GBD 2019 cause hierarchy, headache disorders are classified at Level 3, nested under neurological disorders at Level 2 and non-communicable diseases at Level 1. Under Level 4, headache disorders are further differentiated into migraine and TTH, with no subsequent subdivisions (15). A migraine is a primary headache disorder often characterized by recurrent moderate or severe unilateral pulsatile headaches. The International Classification of Headache Disorders, 3rd edition (ICHD-3) provides definitive diagnostic criteria for migraines. If a patient’s symptoms fulfill all five of these major diagnostic criteria, they are diagnosed with a definite migraine (6, 16). However, any headache that meets all but one of these criteria is termed a probable migraine. TTH manifests as a dull, non-pulsatile, diffuse, band-like or vice-like pain, ranging from mild to moderate intensity, usually located in the head or neck. The diagnostic procedure for TTH mirrors that of migraines. Following the ICHD-3, a definite TTH diagnosis is given if a patient’s symptoms align with all five major criteria, while a probable TTH aligns with all but one criterion (6, 16). In our study, we utilized codes from the International Classification of Diseases, 10th revision (ICD-10), specifically G43-G43.919, G44.2-G44.229, and G44.4-G44.41, to denote migraines and TTH (6, 16).

### Statistical analysis

In this study, we examined the repercussions of migraines and TTH on health outcomes. To quantify the extent of these effects, we utilized various metrics such as incidence, prevalence, DALYs, along with their corresponding rates. DALYs, encompassing years of life lost to premature mortality and years lived with disability, serve as a pivotal metric in gaging disease burden and overseeing public health (17). In this study, the disease burden was articulated with 95% uncertainty intervals (UI). For a detailed understanding of the adopted methodology, we refer readers to the relevant literature (6, 14). Given the heterogeneity in age distributions and populations within the GBD dataset, adjusting for disparities in age structures becomes paramount. Utilizing the age-standardized incidence rate (ASIR), age-standardized prevalence rate (ASPR), and age-standardized DALYs rate enhances the comparability across populations with varying age structures and demographic sizes (18). To quantify the trends in ASRs, we employed the estimated annual percentage change (EAPC) (19). A linear regression model was applied to the natural logarithm of the ASRs, represented as y=α+βx+ε with y being the ln(ASRs) and x indicating the calendar year. The EAPC was determined as 100 × (e^β^ − 1), with its 95% confidence interval (CI) derived from the linear regression model (20). The trajectory of the ASR can be elucidated by examining the EAPC in conjunction with its 95% CI. An upward trend in the ASR is inferred when both the EAPC and the lower boundary of its 95% CI exceed zero. On the other hand, a downward trend in the ASR is indicated when both the EAPC and the upper boundary of its 95% CI fall below zero (20). To project the disease burden from 1990 to 2045, we employed a log-linear age-period-cohort model. We harnessed the NORDPRED software package, developed and implemented in the R programming language, which has demonstrated empirical effectiveness in forecasting future trends (21). The extrapolation process entailed extending the data from the three or four most recent 5-year observed periods (subject to data availability). This extension was achieved using a power function designed to stabilize growth. Specifically, the projection featured a linear trend for the final 10 years, but this trend was dampened by 25% in the second prediction period, 50% in the third prediction period, and 75% in both the fourth and fifth prediction periods. The projections were conducted at 5-year intervals (22). In our investigation, we conducted analyzes and generated graphical representations utilizing the R statistical software, specifically, version 4.2.2. A threshold of 0.05 for the two-tailed *p*-value was employed to establish statistical significance.

## Results

### Prevalence

**Figure 1:**
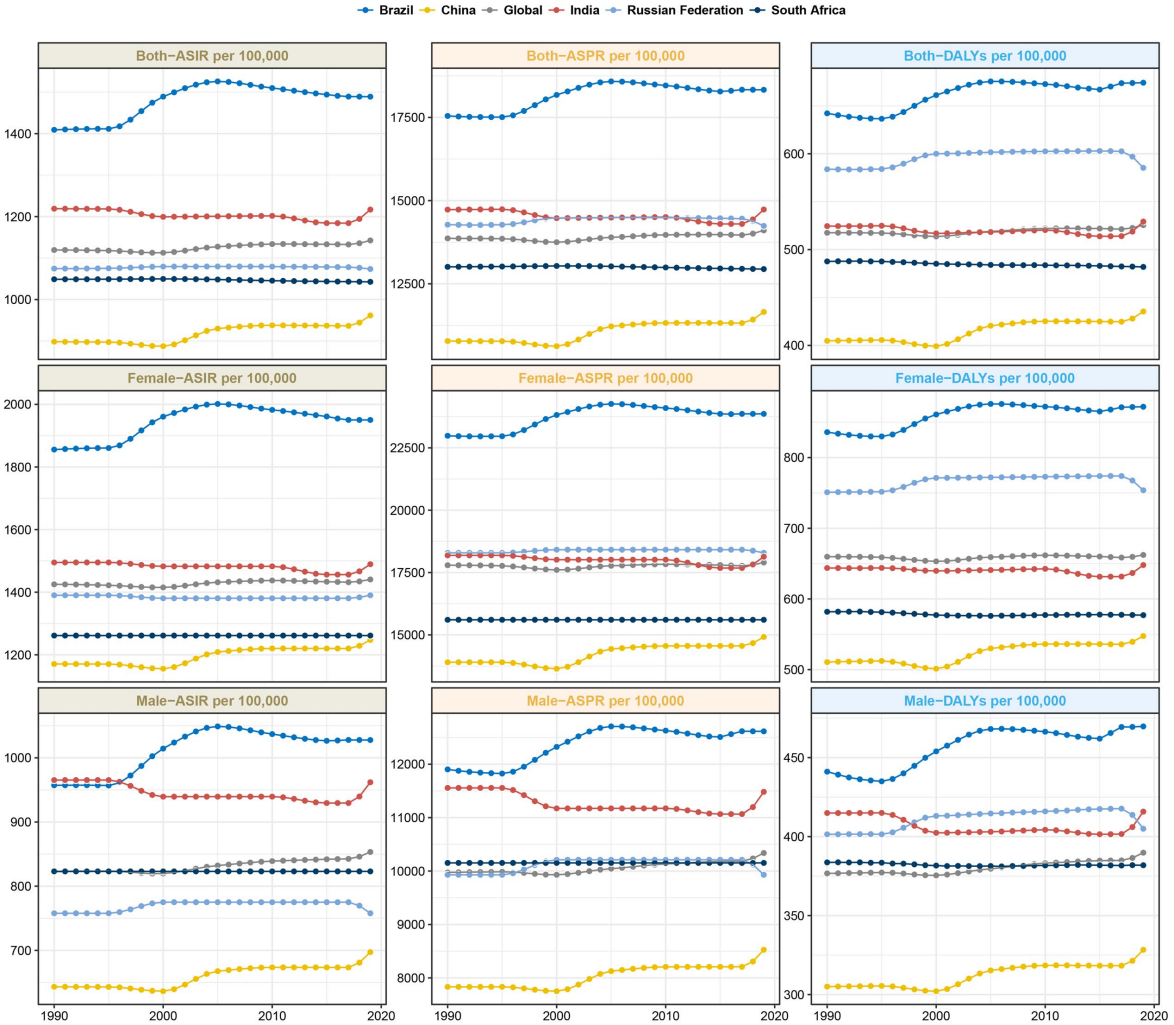
Trends in ASIR, ASPR and age-standardized DALYs rate for migraine in the BRICS countries.

**Figure 2:**
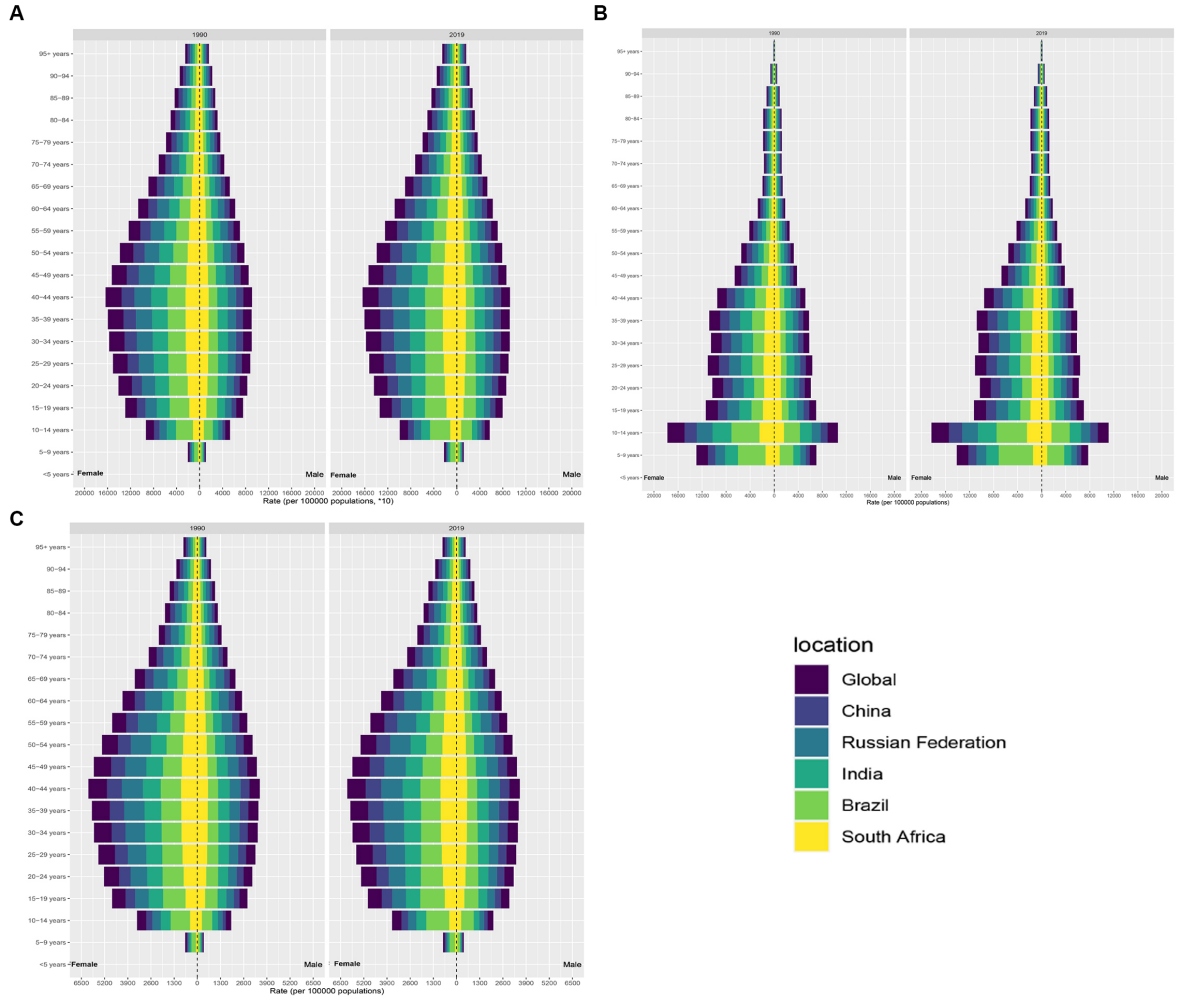
Distribution of migraine prevalence, incidence and DALYs by age in the BRICS countries. **(A)** Distribution of migraine prevalence. **(B)** Distribution of migraine incidence. **(C)** Distribution of migraine DALYs.

As of 2019, Brazil maintained the highest migraine ASPR with 18,331 per 100,000 individuals (Table 1; Figure 1; Supplementary Figure S1A). This represents a notable augmentation of 4.48% since 1990, with an EAPC of 0.19 (95% CI: 0.13 to 0.25) (Table 1; Supplementary Figure S2A). Contrarily, China manifested the lowest ASPR, at 11,654.58 per 100,000 in 2019, marking an 8.1% increase since 1990 (Table 1; Figure 1; Supplementary Figure S1A). Significantly, India’s migraine ASPR saw a minute elevation of 0.02%, marginally shifting from 14,730.88 per 100,000 in 1990 to 14,733.56 per 100,000 in 2019. Concurrently, South Africa documented a subtle decline of 0.51% from 1990 to 2019, transitioning from 13,010.09 to 12,943.04 cases per 100,000 (Table 1; Supplementary Figure S2A). The Russian Federation observed a slight reduction in migraine prevalence, registering a 0.26% decrease over this period (Table 1; Supplementary Figure S2A). In 1990 and 2019, the ASPR for migraine was higher in women than in men, with women reaching the greatest ASPR in the 40–44 age group (Figure 2A).

**Table 1:** Prevalence, incidence and DALYs of migraine between 1990 and 2019 across the BRICS.

Prevalence					
Location	1990		2019		EAPC_95% CI
	Number_95% UI	ASR	Number_95% UI	ASR	
Global	721903028.76 (624861192.87–833350778.44)	13865.65 (12040.68–15907.49)	1128087260.96 (979598830.38–1298138078.06)	14107.26 (12270.27–16239.02)	0.06 (0.04–0.07)
South Africa	4542436.8 (3911043.56–5278121.99)	13010.09 (11282.1–14938.39)	7513716.65 (6513575.55–8667383.82)	12943.04 (11227.03–14848.41)	−0.02 (−0.03–0.02)
India	117242529.7 (101288528.7–135585822.02)	14730.88 (12794.89–16880.16)	213890207.93 (185723716.71–246241418.74)	14733.56 (12833.64–16910.13)	−0.08 (−0.11–0.06)
Brazil	26431396.29 (22724019.38–31449722.09)	17545.55 (15206.83–20508.63)	41255548.03 (35698886.44–48249208.24)	18330.76 (15807.5–21533.92)	0.19 (0.13–0.25)
Russian Federation	22902281.14 (20151388.97–26225171.62)	14277.45 (12507.83–16361.18)	22786027.58 (20050369.28–26143044.69)	14240.37 (12475.82–16324.24)	0.04 (0.02–0.06)
China	131110516.56 (113257174.74–150528419.77)	10780.33 (9423.25–12406.62)	188932055.21 (164819236.56–219200791.92)	11654.58 (10125.14–13464.62)	0.28 (0.23–0.33)
Incidence					
Global	62585283.35 (54459799.28–70979260.25)	1119.53 (977.26–1262.34)	87648968.88 (76635688.35–98654601.92)	1142.54 (995.9–1289.44)	0.08 (0.06–0.09)
South Africa	419227.39 (361434.79–472486.59)	1048.93 (916.37–1174.69)	606270.42 (529330.69–680997.44)	1042.74 (911.08–1167.69)	−0.02 (−0.03--0.02)
India	11150866.39 (9734850.89–12611422.78)	1219.13 (1071.44–1369.09)	17931770.89 (15751990.59–20086848.35)	1216.95 (1065.76–1361.03)	−0.08 (−0.11–0.06)
Brazil	2373719.59 (2049527.4–2717862.08)	1409.16 (1231.46–1595.13)	3023236.64 (2663324.19–3393363.75)	1489.15 (1290.61–1698.33)	0.24 (0.15–0.32)
Russian Federation	1585411.91 (1398185.07–1774270.33)	1074.69 (942.68–1203.72)	1416866.48 (1258455.26–1582178.46)	1073.63 (941.88–1202.44)	0.01 (0–0.02)
China	11310604.65 (9850594.34–12745865.47)	898.39 (783.14–1004.15)	12939764.57 (11463448.8–14485073.27)	961.69 (845.91–1079.18)	0.24 (0.2–0.29)
DALYs					
Global	26863345.21 (3969239.12–61445234.81)	517.58 (81.95–1169.12)	42077665.9 (6418383.33–95645211.01)	525.54 (78.79–1193.99)	0.05 (0.04–0.07)
South Africa	168966.21 (27124.27–378298.77)	487.67 (86.83–1079.34)	279238.11 (48355.51–615375.35)	481.93 (85.88–1066.64)	−0.04 (−0.05–0.04)
India	4173576.79 (474984.49–9595042.5)	524.49 (67.03–1188.11)	7687692.52 (937248.14–17514317.34)	529.22 (68.06–1200.93)	−0.05 (−0.07–0.02)
Brazil	966754.65 (91209.1–2308815.21)	642.17 (71.15–1509.67)	1517871.42 (172802.03–3578740.62)	674.12 (71.77–1605.15)	0.21 (0.16–0.27)
Russian Federation	945012.97 (284155.43–1947330.76)	583.83 (168.1–1209.75)	957458.96 (310326.56–1959392.92)	585.28 (168.67–1210.94)	0.1 (0.06–0.14)
China	4920025.73 (776816.24–11081988.14)	404.81 (66.13–907.01)	7089416.95 (1130495.63–16097219.86)	435.42 (63.68–991.8)	0.27 (0.22–0.32)

**Figure 3:**
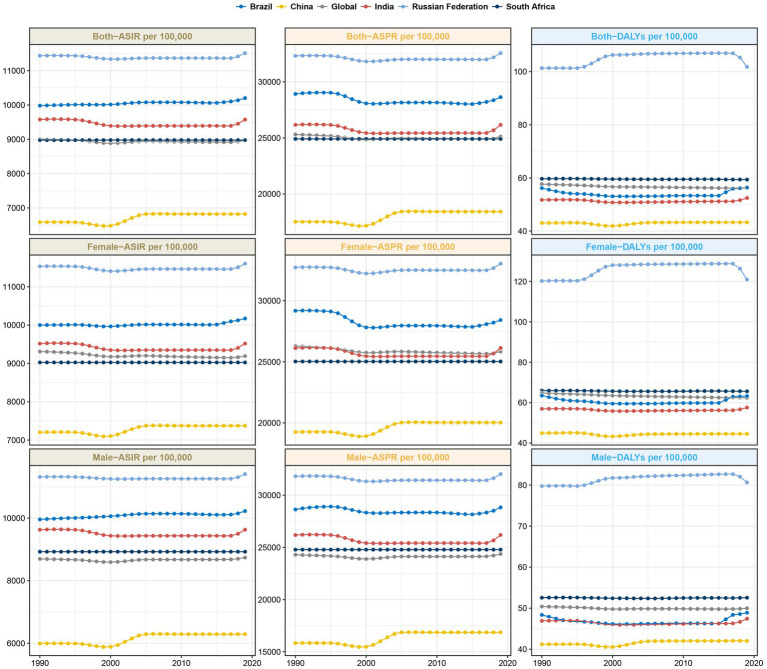
Trends in ASIR, ASPR and age-standardized DALYs rate for TTH in the BRICS countries.

**Figure 4:**
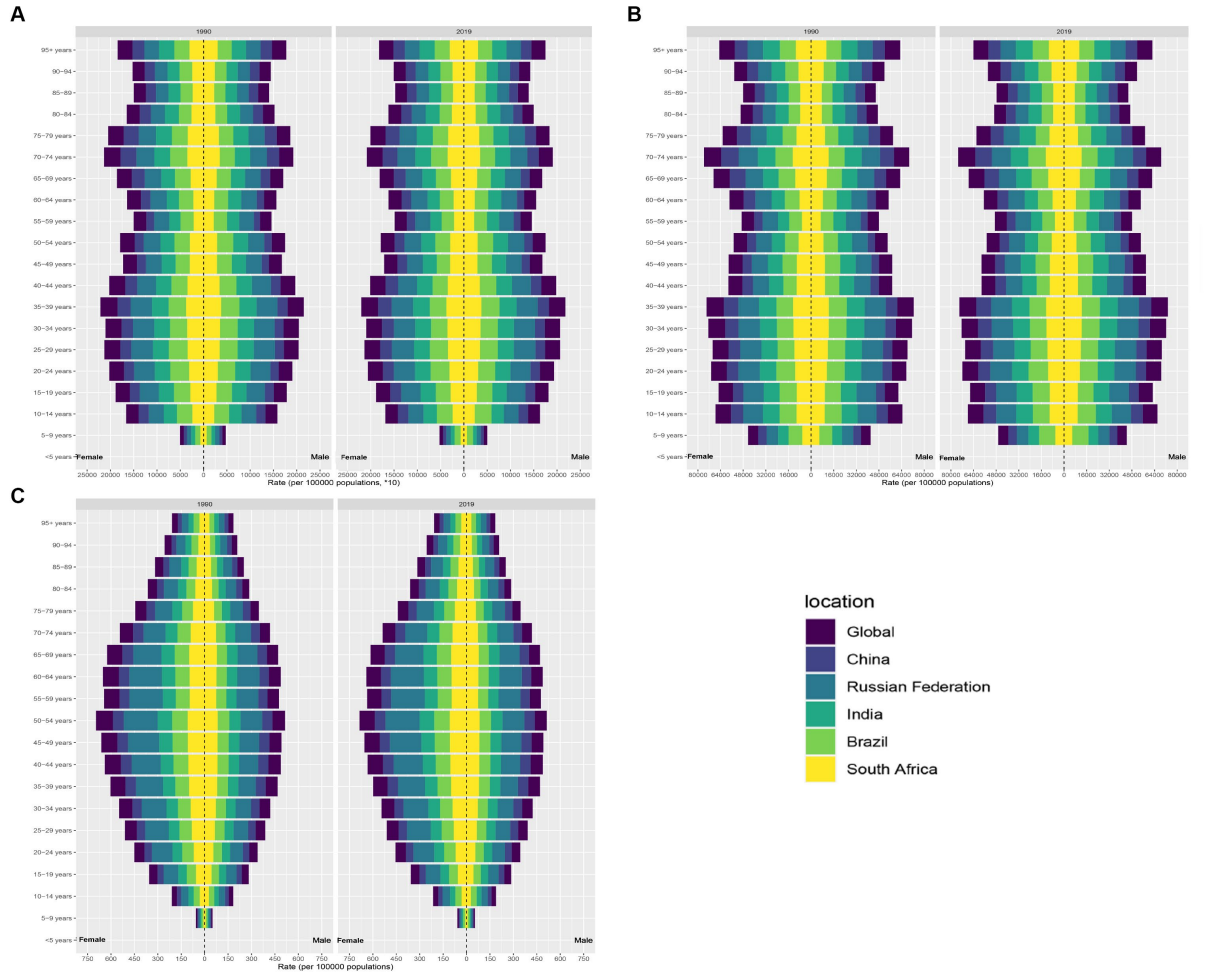
Distribution of TTH prevalence, incidence and DALYs by age in the BRICS countries. **(A)** Distribution of migraine prevalence. **(B)** Distribution of migraine incidence. **(C)** Distribution of migraine DALYs.

In BRICS countries, India shouldered the greatest TTH cases in both 1990 and 2019, with over 200 million estimated cases in 1990 rising sharply to over 374 million by 2019 (Table 2; Supplementary Figure S1B). The ASPR of India also remained similar at approximately 26,160 per 100,000 population between 1990 and 2019 (Table 2; Figure 3; Supplementary Figure S1B). On the other end of the spectrum, South Africa’s ASPR decreased slightly from 24,909 per 100,000 in 1990 to 24,907 per 100,000 in 2019 (Table 2; Figure 3). In China, prevalent cases rose substantially from 208 million to 282 million between 1990 and 2019. Over the same period, ASPR increased slightly from 17,515.14 (95% UI 15,462.06-19,823.37) to 18,423.96 (95% CI 16,133.74-20,802.27) per 100,000 (Table 2; Figure 3; Supplementary Figure S2B). The EAPC was 0.27 (95% CI 0.2–0.34) (Table 2; Supplementary Figure S2B). Russia displayed a slight decrease in prevalent cases from 50.9 million to 50.3 million, while ASPR underwent a small increase from 32,319.09 (95% UI 28,783.56-35,980.02) to 32,560.37 (95% UI 29,068.49-36,148.29) per 100,000 (Table 2; Figure 3; Supplementary Figures S1B, S2B). In contrast to migraine, TTH had a relatively equalized ASPR between genders, with the ASPR reaching a maximum in the 35–39 year age group (Figure 4A).

**Table 2:** Prevalence, incidence and DALYs of tension-type headache between 1990 and 2019 across the BRICS.

Location	1990		2019		EAPC_95% CI
	Number_95% UI	ASR	Number_95% UI	ASR	
Prevalence					
Global	1307510960.66 (1142071268.72–1483958173.65)	25306.23 (22263.45–28510.58)	1995172548.87 (1751946845.72–2242204885.35)	25113.49 (22020.81–28316.24)	−0.04 (−0.06–0.02)
South Africa	8701790.73 (7558853.7–9931804.55)	24909.44 (21919.76–27935.22)	14223961.32 (12467288.49–16072441.65)	24907.19 (21907.83–27935.3)	0 (0–0)
India	206091789.07 (179958821.19–233312004.68)	26163.05 (23093.81–29243.82)	374453700.36 (329045841.18–421227522.15)	26160.92 (23090.1–29248.04)	−0.09 (−0.13–0.05)
Brazil	41997742.48 (36637884.14–47724988.97)	28921.2 (25580.72–32317.73)	64976813.92 (57742298.32–72450443.18)	28630.58 (25403.16–32036.69)	−0.1 (−0.15–0.06)
Russian Federation	50943290.87 (45401274.24–56642740.79)	32319.09 (28783.56–35980.02)	50266356.65 (45153199.29–55370197.72)	32560.37 (29068.49–36148.29)	−0.02 (−0.04–0)
China	208458148.46 (182175913.77–238029453.04)	17515.14 (15462.06–19823.37)	282144907.55 (249592452.33–318439040.02)	18423.96 (16133.74–20802.27)	0.27 (0.2–0.34)
Incidence					
Global	472024829.53 (416622250.27–527313846.87)	9006.97 (7976.7–10044.71)	706190113.83 (626723554.98–788575302.43)	8968.18 (7931.86–9990.52)	−0.02 (−0.03–0.01)
South Africa	3220432.21 (2812795.73–3650583.27)	8976.71 (7956.37–10061.54)	5113301.56 (4489739.36–5763598.68)	8975.93 (7952.67–10062.07)	0 (0–0)
India	77821837.26 (67860242.46–87682315.08)	9577.64 (8516.85–10686.42)	136792784.77 (121078647.71–153329091.18)	9575.36 (8517.77–10684.57)	−0.06 (−0.09–0.03)
Brazil	14846780.35 (12909031.5–16798905.07)	9981.64 (8823.98–11166.59)	22655733.63 (20135893.92–25429456.54)	10201.74 (9009.4–11437.75)	0.05 (0.04–0.06)
Russian Federation	17715445.27 (15727723.21–19748996.74)	11436.63 (10152.67–12788.76)	17250979.06 (15308259.57–19346522.36)	11512.04 (10205.5–12898.54)	−0.01 (−0.03–0)
China	78243733.9 (68920461.31–88919364.49)	6585.21 (5855.32–7428.5)	101281418.84 (89412156.8–114335361.11)	6821.71 (6040.1–7655.16)	0.19 (0.14–0.24)
DALYs					
Global	2878103.32 (853081.78–9769273.39)	57.65 (17.73–188.5)	4541688.88 (1395545.97–14981335.61)	56.21 (17–188.51)	−0.09 (−0.1–0.08)
South Africa	19402.35 (5844.32–67980.29)	59.67 (19.03–191.96)	33651.1 (10836.35–112250.71)	59.36 (19.32–195.59)	−0.02 (−0.02−−0.02)
India	386844.01 (102532.61–1573853.84)	51.71 (14.64–194.95)	741392.13 (206386.92–2795445.49)	52.44 (14.74–192.91)	−0.02 (−0.05–0.02)
Brazil	77021.86 (19442.73–318243.16)	56.16 (15.28–213.18)	131845.74 (37108.25–477924.4)	56.35 (15.33–206.68)	0 (−0.08–0.08)
Russian Federation	167808.06 (62283.02–462920.95)	101.35 (36.54–289.91)	176146.19 (66657.91–474213.81)	101.83 (36.66–291.95)	0.17 (0.1–0.24)
China	503667.14 (160841.26–1829777.34)	43.03 (14.01–153.11)	726401.61 (242671.94–2254632.56)	43.26 (13.55–147.86)	0.05 (0.01–0.09)

### Incidence

In an examination of the BRICS nations, India emerged with the most significant migraine incidence cases in 2019, with an estimated 17,931,771 cases (95% UI: 15,751,991-20,086,848) and an ASIR of 1,216.95 per 100,000 (Supplementary Figure S3A). Following closely, China reported the second-largest incidence, totaling approximately 12,939,765 cases (95% UI: 11,463,449-14,485,073), albeit with the lowest ASIR at 961.69 per 100,000 (Supplementary Figure S3A). Brazil registered the third-largest incidence with about 3,023,237 million cases, but displayed the highest ASIR at 1,489.15 per 100,000 (95% UI: 1,290.61-1,698.33). South Africa had the least incidence, accounting for 606,270 cases (95% UI: 529,331-680,997) (Supplementary Figure S3A). When contrasted with the 1990 estimates, Brazil manifested the most substantial upswing in migraine incidence, marked by an EAPC of 0.24 (95% CI: 0.15–0.32) (Supplementary Figure S4A). Conversely, both South Africa and India experienced a marginal downturn (Supplementary Figure S4A). The incidence in the Russian Federation remained comparatively unaltered during this period (as detailed in Table 1). The incidence of migraine in the BRICS is distributed in a “pyramid” pattern across all age groups. The incidence rate is higher in women than in men, with the highest prevalence in the 10–14 years age group (Figure 2B).

Within the BRICS cohort, India reported the most pronounced TTH incidence in 2019, approximating 136,792,785 cases (95% UI: 121,078,648-153,329,091), which corresponds to an ASIR of 9,575.36 per 100,000 population (95% UI: 8,517.77-10,684.57) (Supplementary Figure S3B). China followed with the second-largest incidence at about 101,281,419 cases (95% UI: 89,412,157-114,335,361) and an ASIR of 6,821.71 per 100,000 (Supplementary Figure S3B). Brazil’s data revealed the third-largest incidence with approximately 22,655,734 cases (95% UI: 20,135,894-25,429,457) and an ASIR of 10,201.74 per 100,000 (95% UI: 9,009.4-11,437.75) (Supplementary Figure S3B). Notably, while the Russian Federation exhibited the highest ASIR, 11,512.04 per 100,000 (95% UI: 10,205.5-12,898.54), China presented the lowest at 6,821.71 (95% UI: 6,040.1-7,655.16). Comparative data from 1990 indicate that China experienced the most significant escalation in TTH incidence, marked by an EAPC of 0.19 (95% CI: 0.14–0.24) (Supplementary Figure S4B). Brazil also exhibited a growth trend (Supplementary Figure S4B). In contrast, incidence patterns in India and South Africa remained largely consistent throughout this period (as illustrated in Table 2). The incidence of TTH reaches its maximum in the age group 70–74 years and minimum in the age group 5–9 years (Figure 4B).

### DALYs

As delineated in Table 1 and Supplementary Figure S5, India demonstrated the highest migraine-associated DALYs, registering 7687692.52 (95% UI: 937248.14–17514317.34). In contrast, South Africa reported the minimal count, documenting 279238.11 (95% UI: 48,355.51-615,375.35). On assessing the DALY rate per 100,000 population, Brazil emerged at the pinnacle with 674.12 (95% UI: 71.77–1,605.15), while China presented the lowest rate, at 435.42 (95% UI: 63.68–991.8) (Figure 1). Notably, China and Brazil experienced pronounced escalations in age-standardized DALY rates. Conversely, India evidenced the most substantial reductions (Table 1). From 1990 to 2019, the DALYs rate for migraine was higher for women than for men, reaching a maximum at ages 40–44 (Figure 2C). Turning to Table 2, India again recorded the highest tally of TTH-associated DALYs, amounting to 741,392.13 (95% UI: 206,386.92-2,795,445.49) (Supplementary Figure S5B), while South Africa posted the least at 33,651.1 (95% UI: 10,836.35-112,250.71) (Supplementary Figure S5B). In terms of DALY rate per 100,000 population, the Russian Federation led with 101.83 (95% UI: 36.66–291.95) (Figure 2). However, China depicted the most conservative rate of 43.26 (95% UI: 13.55–147.86) (Figure 2). Remarkably, both China and Brazil showcased upward trajectories in age-standardized DALY rates (Table 2). From 1990 to 2019, the DALYs Rate for TTH is higher for women than for men, reaching a maximum at ages 50–54 and gradually decreasing after ages 60–64 (Figure 4C).

### Drivers of migraine and TTH epidemiology: population growth, aging, and epidemiologic changes

**Figure 5:**
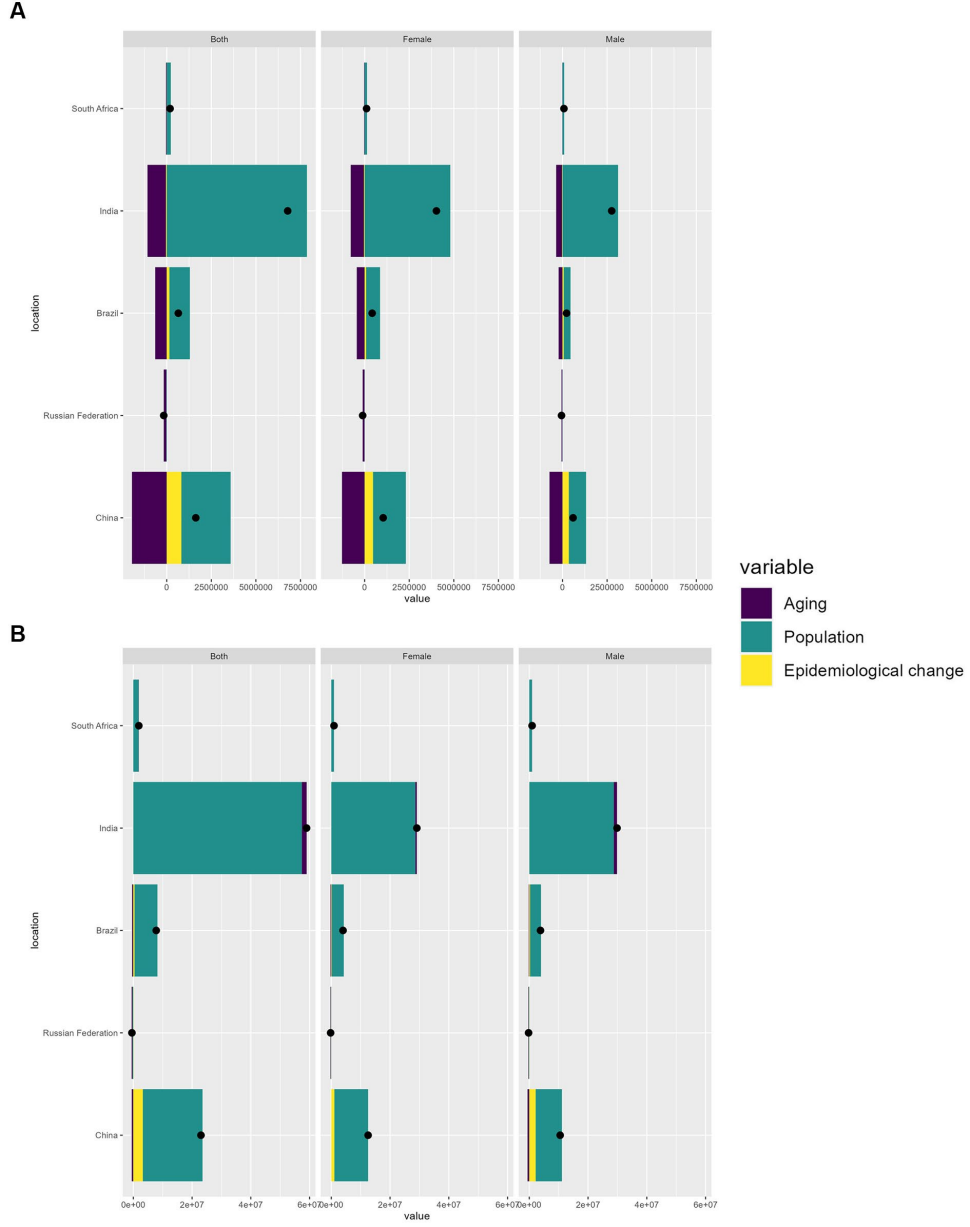
Variations in incidence of migraine **(A)** and TTH **(B)** in BRICS countries from 1990 to 2019, Influenced by population growth, aging, and epidemiological shifts. The black dot denotes the cumulative effect of all three determinants. For each determinant, a positive value signifies an associated rise in prevalence due to that factor, while a negative value suggests a decline in prevalence linked to that specific determinant.

In an effort to delineate the relative impacts of demographic expansion, the aging process, and shifts in epidemiological patterns on the changing landscape of migraine and TTH epidemiology over the last 30 years, we undertook a comprehensive decomposition analysis (23). Overall, significant increases in migraine and TTH prevalence have been observed in all BRICS countries (except Russia), but most notably in India and China, which have seen the largest increases in migraine and TTH prevalence over the past 30 years (Supplementary Figure S6; Tables 3, 4). Between 1990 and 2019, population growth led to a 91.07 and 62.06% increase in the burden of migraine prevalence in India and China, respectively, and a 95.1% increase in the burden of disease in Brazil (Table 3). Whereas population growth remains an important driver of migraine incidence, population aging is a protective factor for migraine incidence (Figure 5; Table 3). In terms of migraine DALYs, population growth is a driver of DALYs in most of the BRICS countries, whereas in the case of population aging it is an important driver of migraine DALYs in Russia, in China, population aging and epidemiological change play a role in migraine DALYs to a similar extent (17.9% VS 20%) (Supplementary Figure S7; Table 3). In TTH, population growth continues to play a major driving role in incidence, prevalence, and DALYs (Figure 5; Table 4; Supplementary Figures S6, S7). In addition, Epidemiological change plays a larger role in migraine and TTH in China compared to other BRICS countries (Tables 3, 4).

**Table 3:** Changes in migraine prevalence, incidence and DALYs number according to population-level determinants and causes from 1990 to 2019.

Location	Measure	Overall difference^a^	Aging^b^	Population^c^	Epidemiological change^d^
South Africa	DALYs	110271.9	14744.2 (13.37%)	98240.86 (89.09%)	−2713.16 (−2.46%)
South Africa	Prevalence	2971279.85	365231.06 (12.29%)	2641662.96 (88.91%)	−35614.18 (−1.2%)
South Africa	Incidence	187043.03	−37728.39 (−20.17%)	228041.91 (121.92%)	−3270.49 (−1.75%)
India	DALYs	3514115.73	323141.44 (9.2%)	3148514.49 (89.6%)	42459.8 (1.21%)
India	Prevalence	96647678.24	8900199.06 (9.21%)	88016860.37 (91.07%)	−269381.19 (−0.28%)
India	Incidence	6780904.51	−1039804.28 (−15.33%)	7860850.98 (115.93%)	−40142.19 (−0.59%)
Brazil	DALYs	551116.77	−28229.72 (−5.12%)	517101.79 (93.83%)	62244.7 (11.29%)
Brazil	Prevalence	14824151.74	−816564.48 (−5.51%)	14097199.31 (95.1%)	1543516.91 (10.41%)
Brazil	Incidence	649517.05	−647881.91 (−99.75%)	1150877.83 (177.19%)	146521.13 (22.56%)
Russian Federation	DALYs	12445.99	22747.08 (182.77%)	−12370.35 (−99.39%)	2069.26 (16.63%)
Russian Federation	Prevalence	−116253.56	257865.69 (−221.81%)	−297082.16 (255.55%)	−77037.09 (66.27%)
Russian Federation	Incidence	−168545.42	−147754.04 (87.66%)	−19510.93 (11.58%)	−1280.46 (0.76%)
China	DALYs	2169391.22	388404.07 (17.9%)	1347025.53 (62.09%)	433961.62 (20%)
China	Prevalence	57821538.64	9520867.8 (16.47%)	35883777.53 (62.06%)	12416893.32 (21.47%)
China	Incidence	1629159.93	−1951920.22 (−119.81%)	2762704.57 (169.58%)	818375.58 (50.23%)

a Change number between year 2019 and 1990.

b Change number due to change in the age structure.

c Change number due to change in population number.

d Change number due to epidemiologic changes. Epidemiologic changes refer to the number change when age structure and population hold constant.

**Table 4:** Changes in TTH prevalence, incidence and DALYs number according to population-level determinants and causes from 1990 to 2019.

Location	Measure	Overall difference^a^	Aging^b^	Population^c^	Epidemiological change^d^
South Africa	DALYs	14248.75	2819.96 (19.79%)	11567.5 (81.18%)	−138.7 (−0.97%)
South Africa	Prevalence	5522170.59	493436.51 (8.94%)	5030147.99 (91.09%)	−1413.9 (−0.03%)
South Africa	Incidence	1892869.35	58975.33 (3.12%)	1834357.86 (96.91%)	−463.83 (−0.02%)
India	DALYs	354548.12	49005.27 (13.82%)	297958.66 (84.04%)	7584.19 (2.14%)
India	Prevalence	168361911.3	13928799.51 (8.27%)	154444335.95 (91.73%)	−11224.17 (−0.01%)
India	Incidence	58970947.51	1643822.16 (2.79%)	57347171.94 (97.25%)	−20046.58 (−0.03%)
Brazil	DALYs	54823.88	11525.51 (21.02%)	42952.86 (78.35%)	345.52 (0.63%)
Brazil	Prevalence	22979071.43	1236167.13 (5.38%)	22258422.06 (96.86%)	−515517.75 (−2.24%)
Brazil	Incidence	7808953.28	−419249.23 (−5.37%)	7816285.52 (100.09%)	411916.98 (5.27%)
Russian Federation	DALYs	8338.13	9887.1 (118.58%)	−2236.84 (−26.83%)	687.87 (8.25%)
Russian Federation	Prevalence	−676934.22	−345532.15 (51.04%)	−658101.49 (97.22%)	326699.42 (−48.26%)
Russian Federation	Incidence	−464466.21	−332138.94 (71.51%)	−227346.36 (48.95%)	95019.09 (−20.46%)
China	DALYs	222734.47	81737.55 (36.7%)	138307.72 (62.1%)	2689.2 (1.21%)
China	Prevalence	73686759.09	5316694.88 (7.22%)	55366254.17 (75.14%)	13003810.05 (17.65%)
China	Incidence	23037684.94	−531625.63 (−2.31%)	20313793.67 (88.18%)	3255516.9 (14.13%)

a Change number between year 2019 and 1990.

b Change number due to change in the age structure.

c Change number due to change in population number.

d Change number due to epidemiologic changes. Epidemiologic changes refer to the number change when age structure and population hold constant.

### Future burden of migraine and TTH

**Figure 6:**
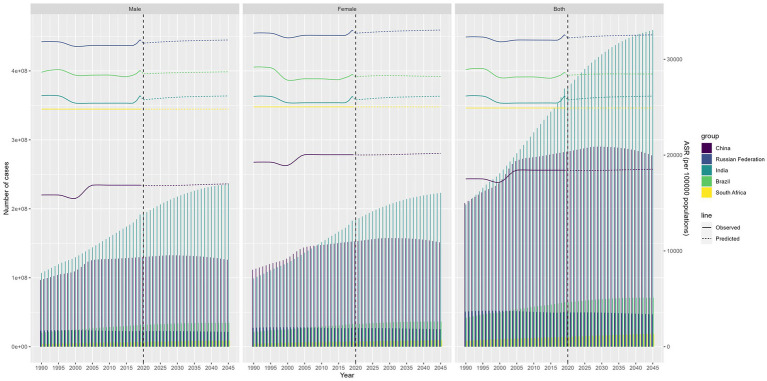
Future forecasts of BRICS countries in TTH prevalence.

**Figure 7:**
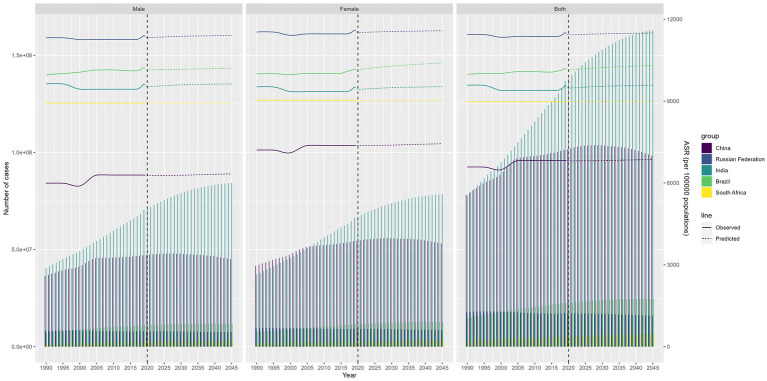
Future forecasts of BRICS countries in TTH incidence.

In Figures 6, 7, we present the anticipated trajectories of TTH prevalence and incidence within the BRICS nations. The data delineate a subtle augmentation in TTH prevalence and ASPR in India, whereas the trajectories in the remaining nations remain largely unchanged. The patterns observed for prevalence and ASIR exhibit congruence. Pertaining to migraine prevalence, India is projected to experience a modest rise in affected individuals, even as the ASPR demonstrates a successive decline. Conversely, Russia is anticipated to persist with its extant decremental trend, while China’s ASPR is forecasted to ascend. In the context of incidence, both the ASIR and the aggregate incidence cases in India are predicted to undergo a reduction. China’s ASIR is expected to witness a marked surge, Russia’s incidence cases are projected to diminish annually, and the ASIR in Russia is likely to maintain its current stability, as illustrated in Supplementary Figures S8, S9.

## Discussion

This comprehensive analysis of GBD 2019 offers salient insights into the evolving landscape of migraine and TTH within the BRICS nations over the past three decades. Our findings highlight the considerable disease burden imposed by these conditions, underpinned by their surging prevalence and disability. In 2019, India and China collectively accounted for over 35% of global migraine cases, while also contributing substantially to TTH prevalence. However, the trends in disease metrics have followed starkly divergent trajectories across the BRICS countries.

India is besieged by a mounting affliction of migraine and TTH, fueled chiefly by unfettered population growth. In 2019, the prevalence of migraine and TTH in India reached 213890207.93 and 374453700.36, respectively, (in 1990, the prevalence of migraine and TTH was 117242529.7 and 206091789.07 respectively). However, when adjusted for population expansion, the age-standardized prevalence rates reveal that the actual disease burden has remained largely unaltered for both conditions. This stagnation indicates that the proliferation of cases is predominantly attributable to demographic expansion rather than intensifying disease epidemiology. Analogously, DALY and incidence also highlight population growth as the cardinal driver, while epidemiological changes exert negligible influence.

In comparison, China presents a contrasting narrative, characterized by ascending ASPR and ASIR for migraine and TTH. The escalation is propelled not merely by population growth but also by epidemiological change and aging. The mounting burden parallels the epidemiological transition accompanying China’s socioeconomic evolution. Rapid urbanization and industrialization have precipitated lifestyle changes, including sedentary habits, stress, obesity, and deteriorating sleep patterns, all of which exacerbate headache disorders (11).

Brazil possesses the highest age-standardized migraine prevalence globally, afflicting over 18% of the populace. However, the uptrend in prevalence and incidence has recently plateaued, potentially indicating saturation. In terms of disability, Brazil exhibits the highest migraine DALY rates within BRICS, congruent with Evidence highlighting the nation’s immense migraine-associated disability. Unlike China and India, Brazil is undergoing an advanced stage epidemiological transition, which may explicate the stabilization in prevalence.

Among the BRICS countries with the highest migraine burden, Brazil has the highest ASPR, ASIR, afflicting over 18% of the populace (12). In addition, according to the results of our projections, the number of TTH in Brazil will continue to increase, suggesting a great pressure and urgency in the field of headache disease prevention and treatment in Brazil in the future.

Conversely, evidence from South Africa and Russia reveals largely unchanging or decrementing patterns for migraine and TTH over the past three decades. Plausible factors responsible for Russia’s dynamics include the impacts of anti-migraine medications and the epidemiological transition nearing completion.

In projecting future trends, our analysis predicts a continuation of current trends in migraine prevalence and incidence in most countries, with the exception of China. Age-standardized migraine prevalence and incidence are projected to increase sequentially in China, and migraine prevalence cases continues to increase in India, with India continuing to have the highest prevalence among the BRIC countries. As for TTH, it is predicted that the number of people with TTH continues to increase rapidly in India, while the rest of the countries remain relatively stable.

Our study has several strengths. We provide a comprehensive epidemiological profile of migraine and TTH encompassing metrics of prevalence, incidence, and DALYs within BRICS nations. Moreover, we qualitatively explore the specific contributions of demographic and epidemiological factors responsible for the evolving disease burden. The exhaustive GBD database ensures rigor and reliability of findings.

### Limitations

Our study primarily analyzed the trends associated with two main types of headaches: migraines and TTH, leaving out trends for other headache disorders. It’s pivotal to emphasize that the foundation of our research is based on the GBD studies, which do not derive from primary data sources. This is particularly concerning as many countries, especially those in the Global South and certain conflict-ridden African nations, lean heavily on unverified estimates due to challenges they face in resources, expertise, and infrastructure essential for exhaustive headache epidemiology on a large scale. Such a dependence might infuse our results with biases and potential inaccuracies. Further complicating the issue is the fact that diagnostic criteria for migraines and TTH can differ, potentially leading to inconsistent incidence rates. These disparities might be magnified by cultural, regional, and methodological differences, with regional and cultural interpretations affecting the consistency in case identification. It’s worth noting that the evident surge in headache burden since the early 1990s might be influenced by improvements in diagnosis and heightened public awareness, factors our study does not distinctly account for. Without dedicated research dissecting this influence, our conclusions could be somewhat skewed. Even though we have endeavored to harmonize methodological differences across various studies, it’s pertinent to mention that some of the variations we observed might arise from measurement errors or intrinsic methodological biases, as opposed to genuine differences.

## Conclusion

In the BRICS nations, headache disorders present a significant burden, as evidenced by our findings. Both migraine and TTH persist as prevalent conditions, affecting a vast number of individuals and resulting in notable disability. Notably, the epidemiological transition varies across these countries, influencing the temporal patterns of these disorders. Specifically, India and China demonstrate increasing trends, driven by demographic growth and shifts in lifestyle. Such insights are instrumental in formulating healthcare policies and allocating resources in BRICS nations, aligning with their unique epidemiological profiles. Our projections further aid in healthcare strategizing, forecasting upcoming needs and challenges. Additionally, our study elucidates the intricate nexus between demographic factors and epidemiology in determining disease prevalence. This interrelation is pivotal and should be factored into interventions aimed at specific risk determinants. In summation, given the profound societal ramifications, there is a compelling case for elevating the priority of headache disorders in the national health strategies of BRICS nations. Comprehensive policies that encompass awareness, prevention, diagnosis, and management hold the potential to significantly mitigate the burden of migraine and TTH.

## Data availability statement

The original contributions presented in the study are included in the article/Supplementary material, further inquiries can be directed to the corresponding authors.

## Author contributions

Y-jZ: Conceptualization, Investigation, Supervision, Writing – original draft. X-yL: Conceptualization, Investigation, Supervision, Writing – original draft. Z-lG: Conceptualization, Investigation, Writing – original draft.
