# Supplementary figures and images for: Temporal trends of migraine and tension-type headache burden across the BRICS: implications from the Global Burden of Disease study 2019
Source: Front Neurol. 2023 Dec 22;14:1307413. doi: 10.3389/fneur.2023.1307413 (PMC10771321; doi:10.3389/fneur.2023.1307413)

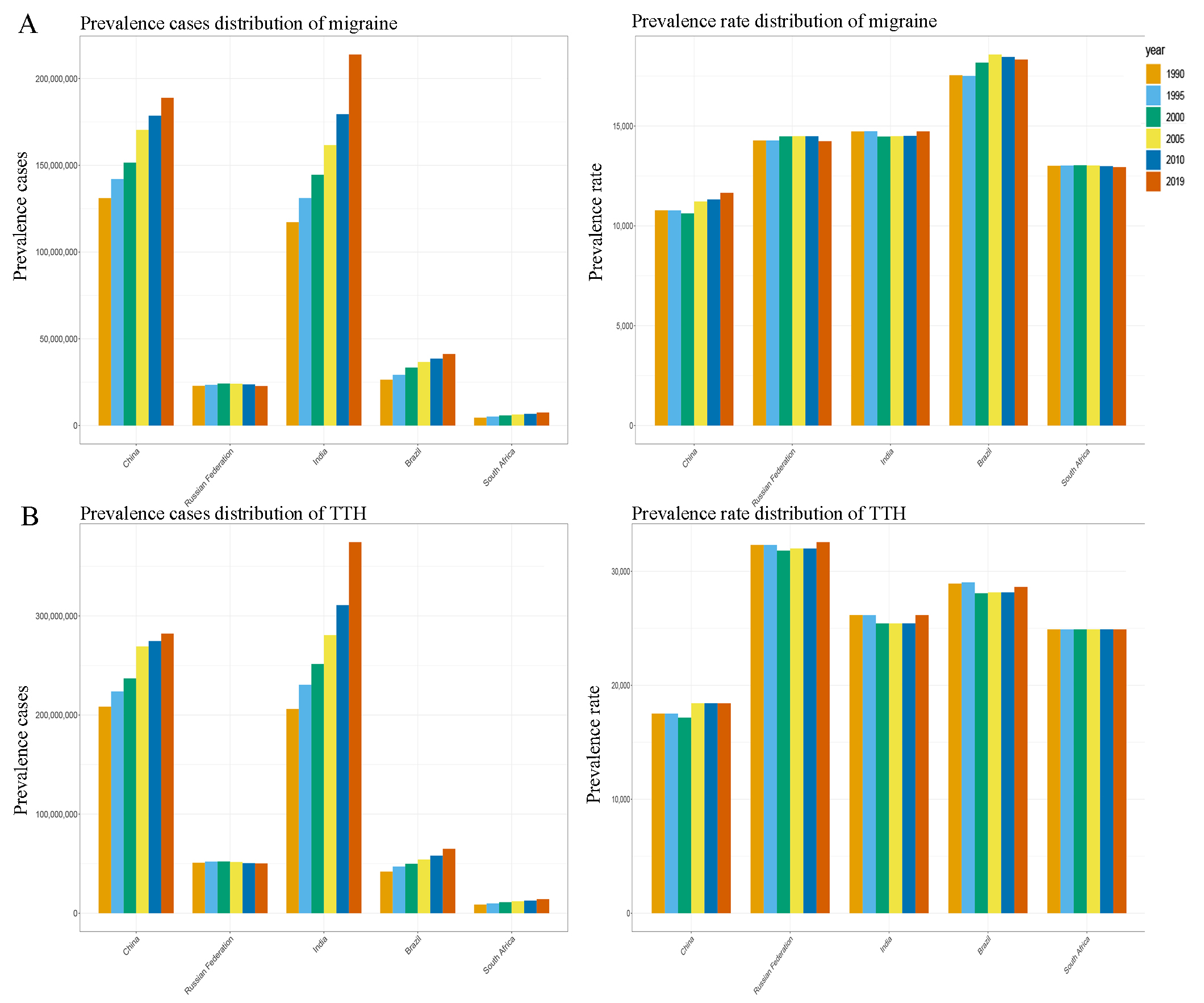

Supplement: Supplementary file 1 [file Image_1.JPEG]

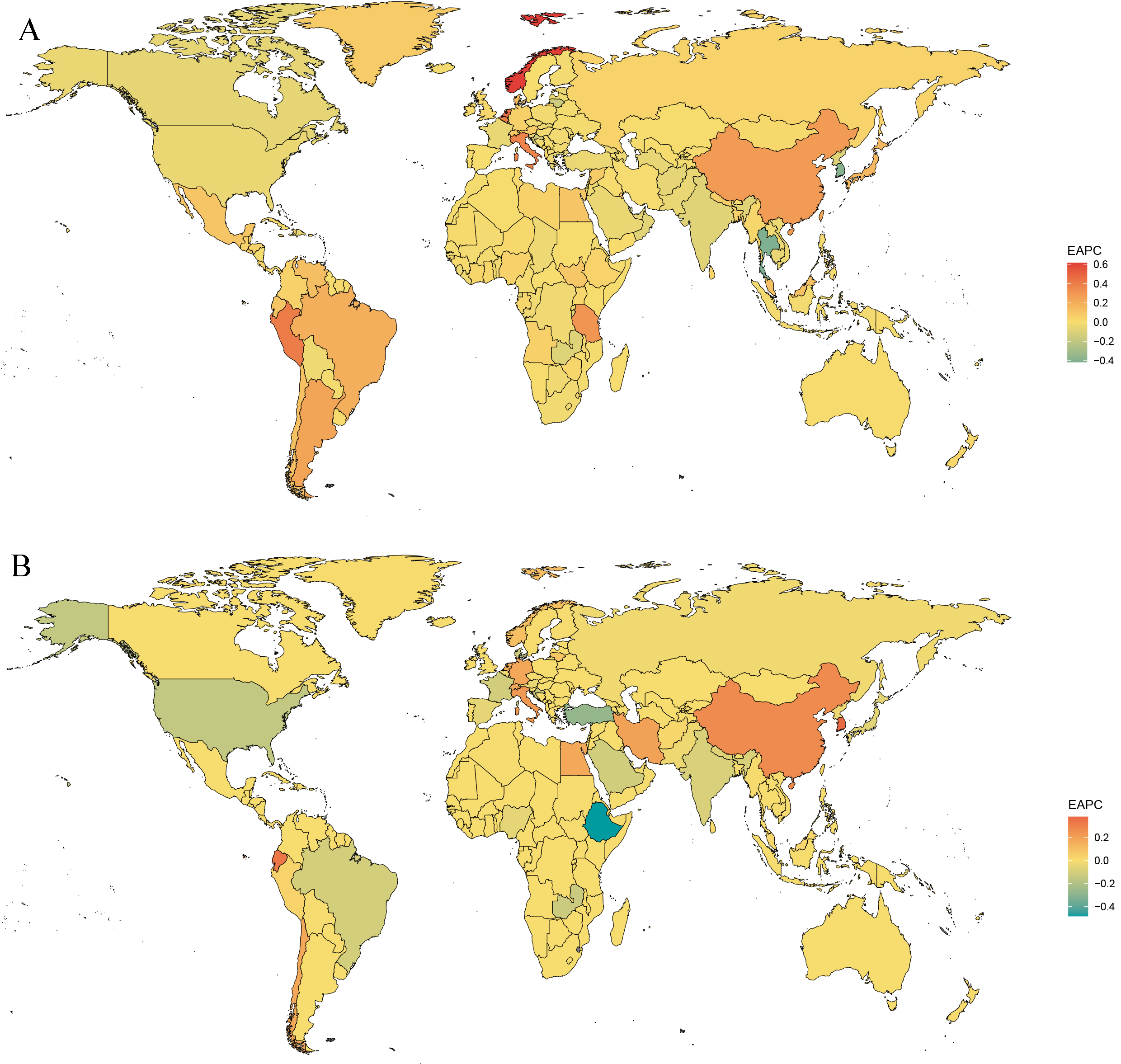

Supplement: Supplementary file 2 [file Image_2.JPEG]

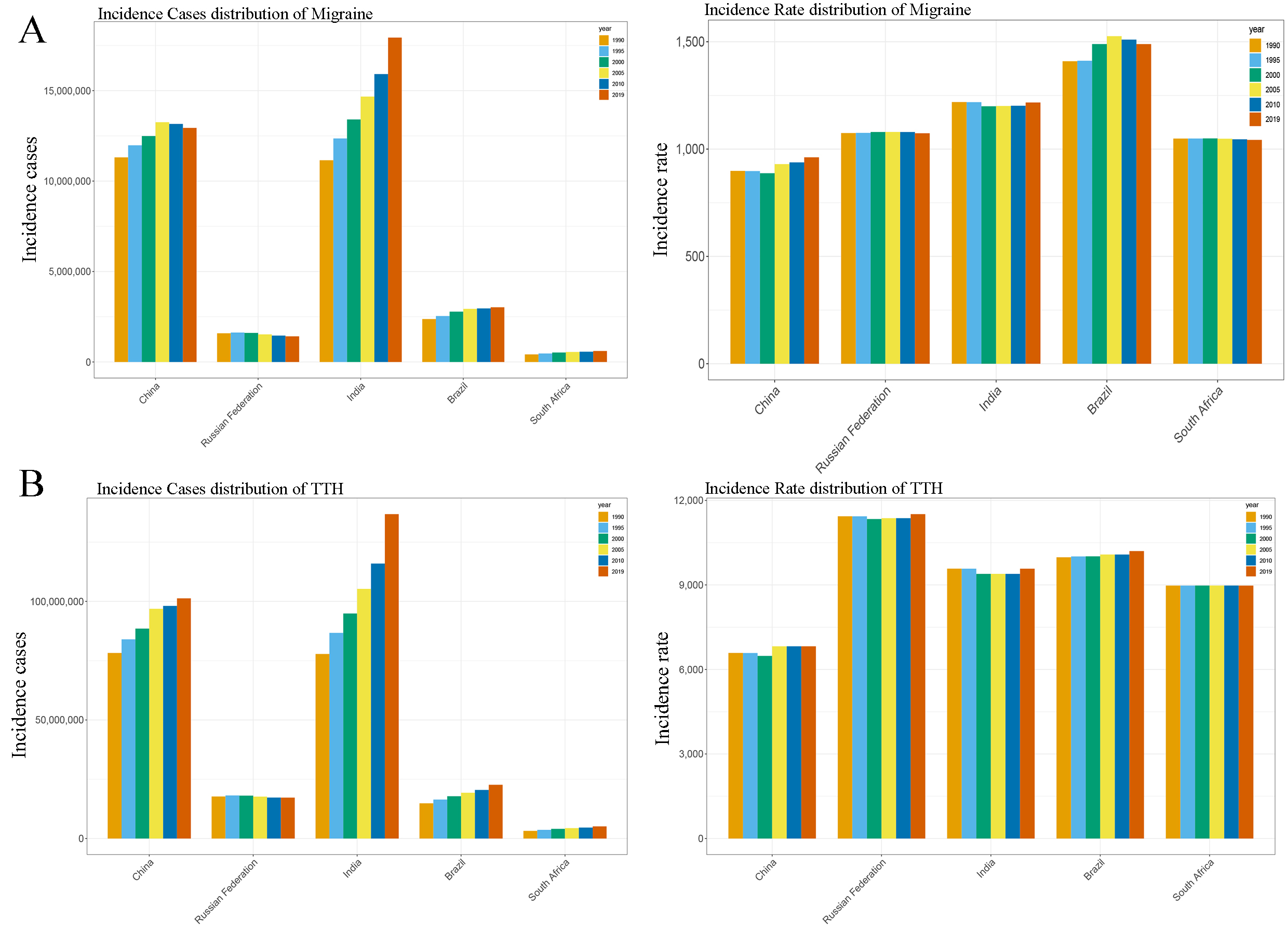

Supplement: Supplementary file 3 [file Image_3.JPEG]

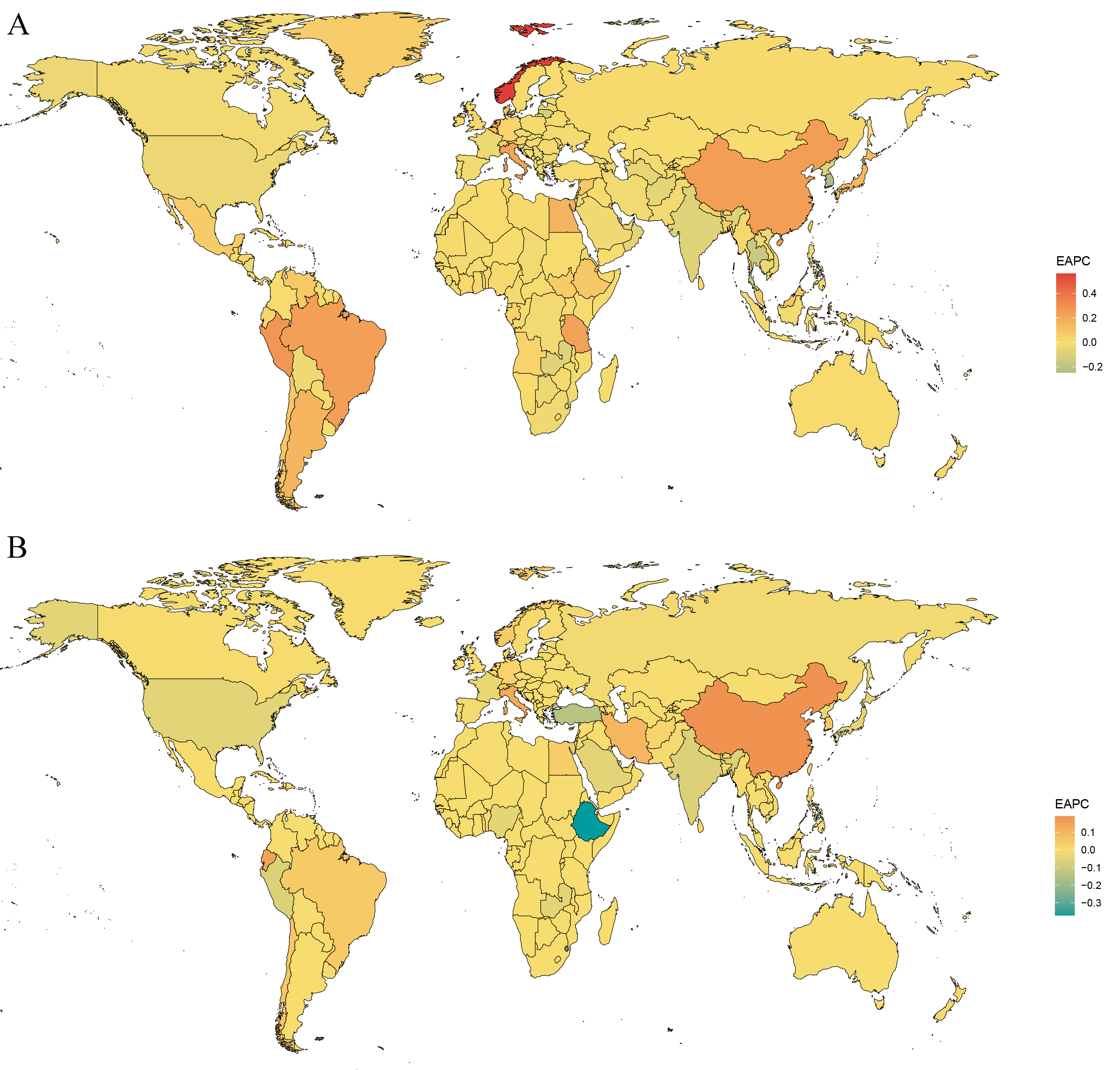

Supplement: Supplementary file 4 [file Image_4.JPEG]

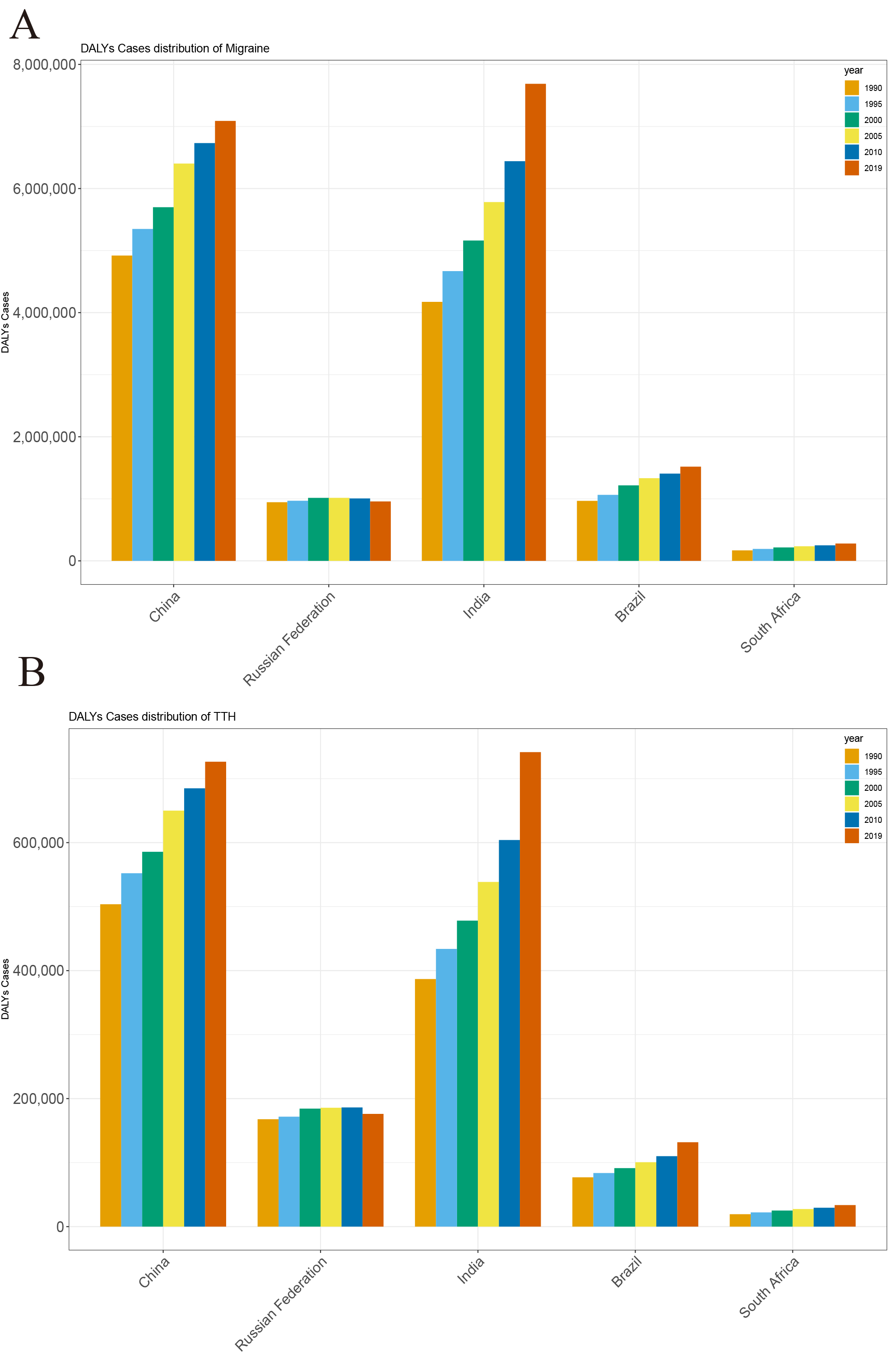

Supplement: Supplementary file 5 [file Image_5.JPEG]

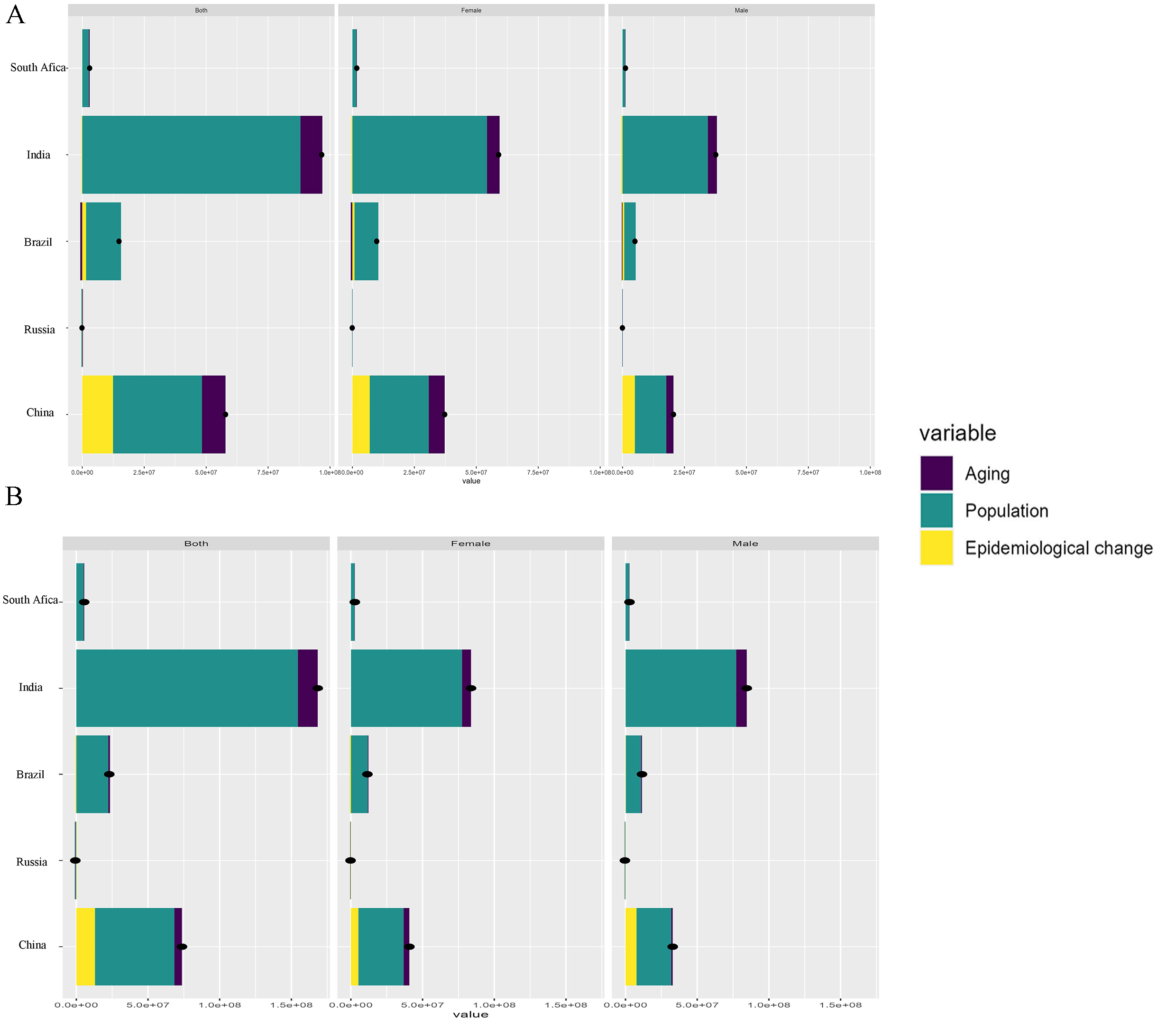

Supplement: Supplementary file 6 [file Image_6.JPEG]

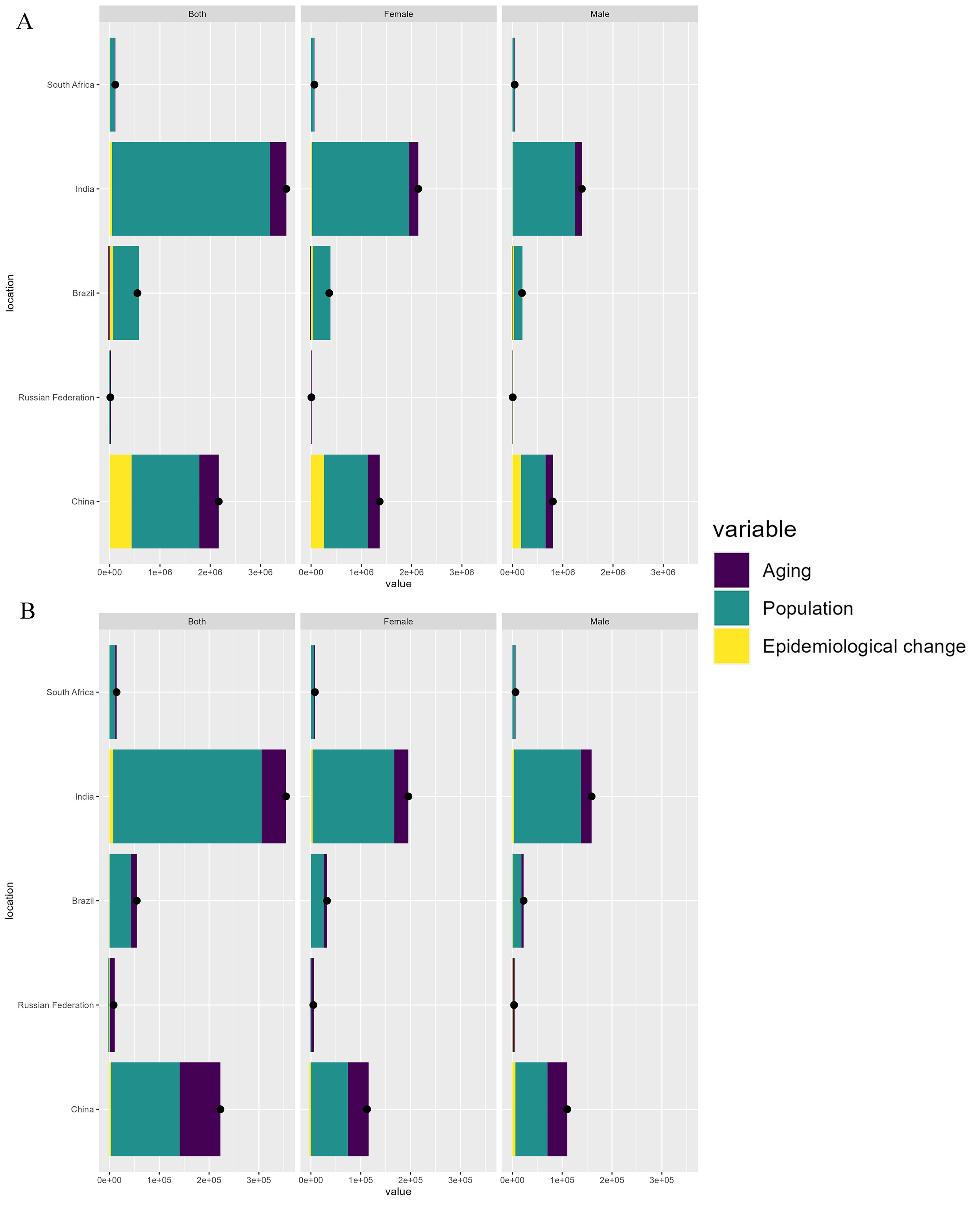

Supplement: Supplementary file 7 [file Image_7.JPEG]

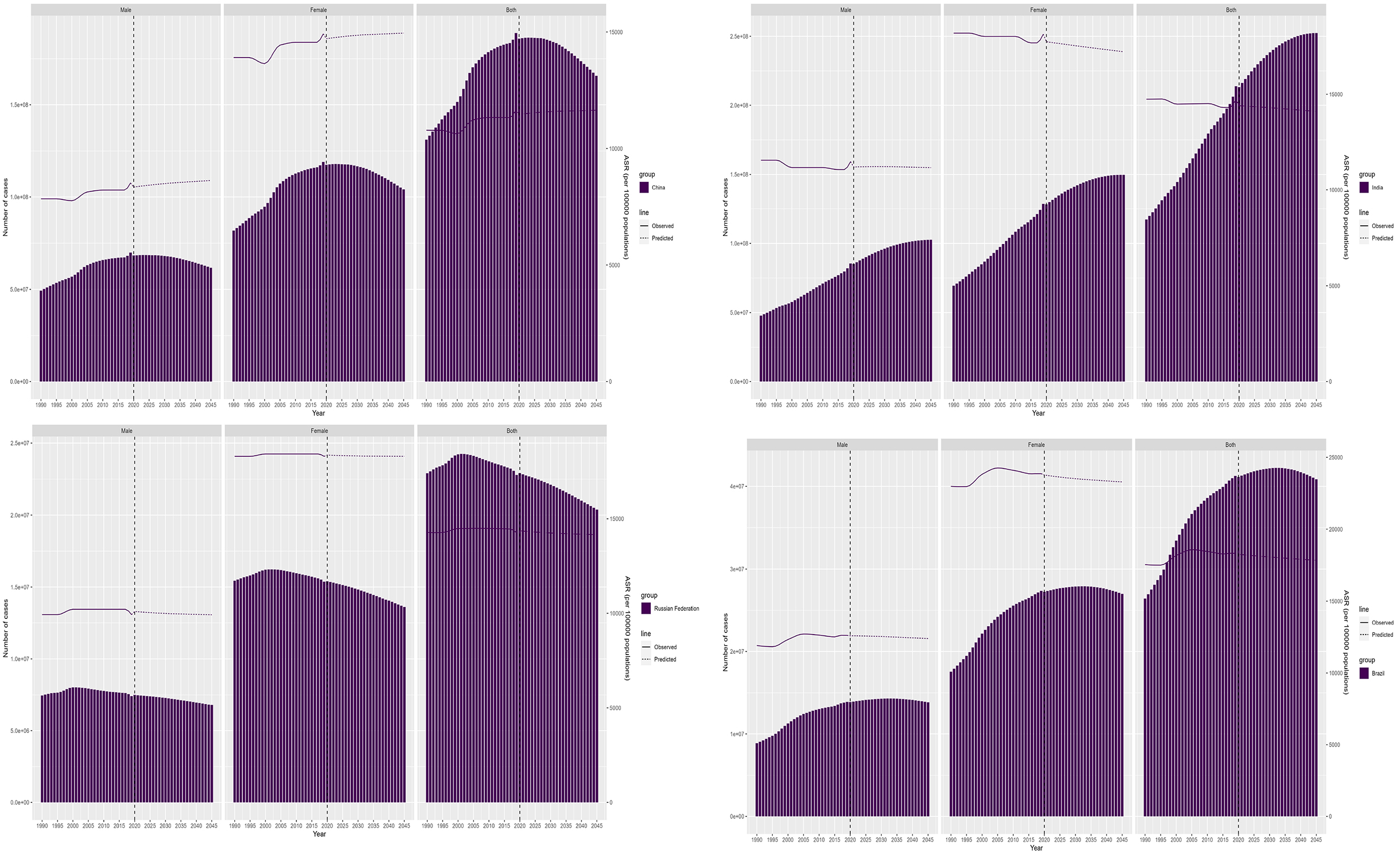

Supplement: Supplementary file 8 [file Image_8.JPEG]

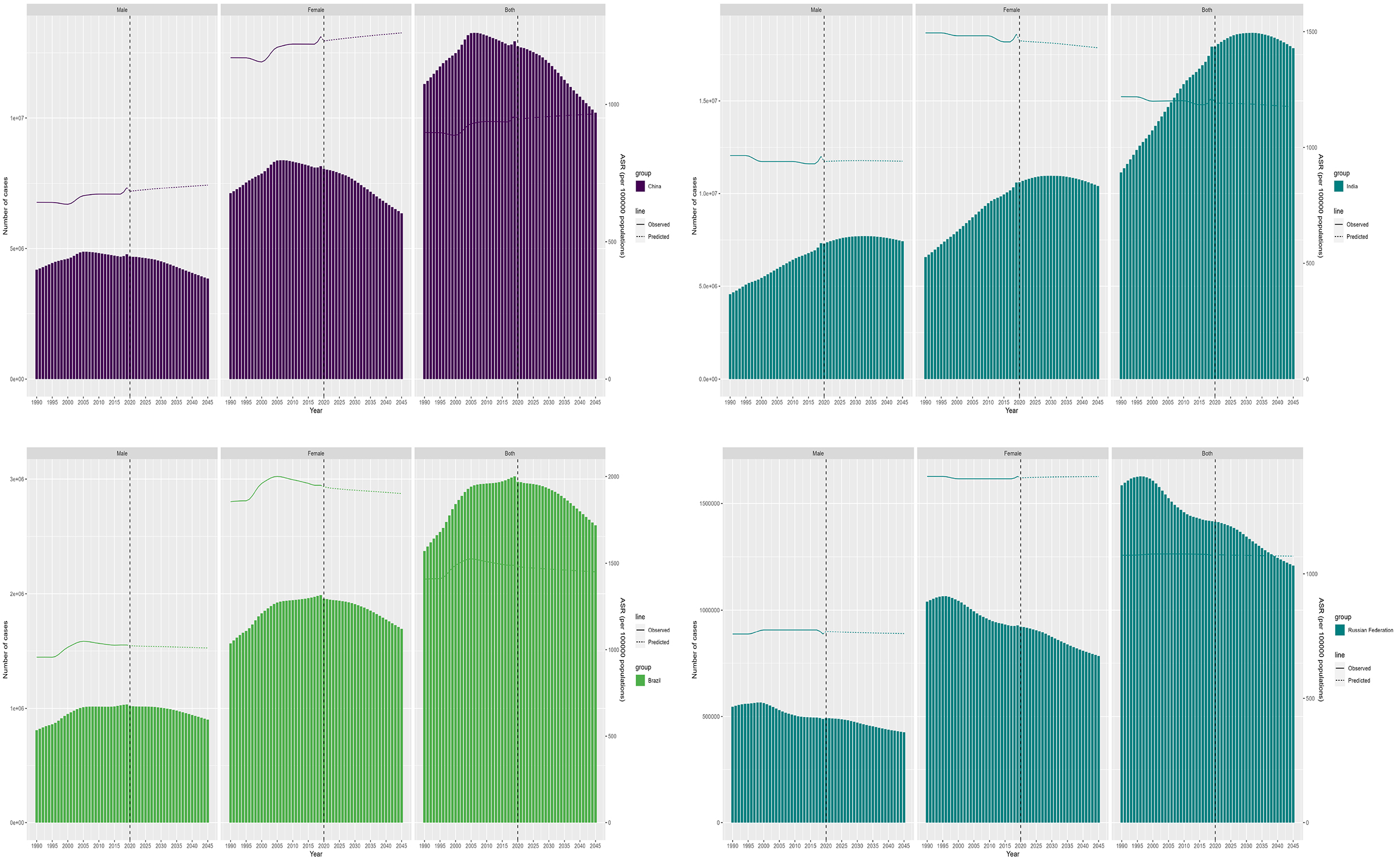

Supplement: Supplementary file 9 [file Image_9.JPEG]
